# A Longitudinal Follow-Up Study of Intellectual Function in Duchenne Muscular Dystrophy over Age: Is It Really Stable?

**DOI:** 10.3390/jcm12020403

**Published:** 2023-01-04

**Authors:** Daniela P. R. Chieffo, Federica Moriconi, Marika Pane, Simona Lucibello, Elisabetta Ferraroli, Giulia Norcia, Martina Ricci, Anna Capasso, Gianpaolo Cicala, Bianca Buchignani, Giorgia Coratti, Costanza Cutrona, Monia Pelizzari, Claudia Brogna, Jos G. M. Hendriksen, Francesco Muntoni, Eugenio Mercuri

**Affiliations:** 1 Psychology Unit, Fondazione Policlinico Universitario Agostino Gemelli IRCCS, 00168 Rome, Italy; 2 Department of Life Science and Public Health, Pediatric Neurology, Università Cattolica del Sacro Cuore, 00168 Rome, Italy; 3 Centro Clinico Nemo, Fondazione Policlinico Universitario Agostino Gemelli IRCCS, 00168 Rome, Italy; 4 Department of Clinical and Experimental Medicine, Università di Pisa, 56126 Pisa, Italy; 5 Department of Neurology, Maastricht University Medical Center, 6229 HX Maastricht, The Netherlands; 6 Dubowitz Neuromuscular Centre, UCL Great Ormond Street Institute of Child Health, London WC1N 1EH, UK; 7 NIHR Great Ormond Street Hospital Biomedical Research Centre, Great Ormond Street Institute of Child Health, London WC1N 1EH, UK; 8 Great Ormond Street Hospital Trust, London WC1N 1EH, UK

**Keywords:** neuromuscular disorders, pediatric, progressive muscular dystrophy, cognitive, neurobehavioral, ADHD

## Abstract

The aim of the study was to retrospectively evaluate the consistency of longitudinal findings on intellectual functioning in DMD boys and their relationship to behavioral and neuropsychiatric difficulties. The cohort included 70 patients of age 3 to 17 years with at least two assessments using the Wechsler scales. CBCL and clinical observation of behavior were also performed. Changes in total intelligence quotient were interpreted as stable or not stable using the reliable-change method. On the first assessment 43/70 had normal quotients, 18 borderline, 5 mild, and 4 moderate intellectual disability, while 27/70 had no behavioral disorders, 17 had abnormal CBCL, and 26 patients had clear signs of attention deficits despite normal CBCL. The remaining seven were untestable. The mean total intelligence quotient change in the cohort was −2.99 points (SD: 12.29). Stable results on TIQ were found in 63% of the paired assessments. A third of the consecutive cognitive assessments showed a difference of more than 11 points with changes up to 42 points. Boys with no behavioral/attention disorder had smaller changes than those with attention (*p* = 0.007) and behavioral disorders (*p* = 0.002). Changes in IQ may occur in Duchenne and are likely to be associated with behavioral or attention deficits.

## 1. Introduction

In a pioneering work published in 1981, Dubowitz and Leibovitz reported that cognitive abilities in Duchenne muscular dystrophy (DMD) boys were about 1 standard deviation (SD) below the mean, with better scores on performance than on verbal tasks [1]. This cross-sectional study also reported, for the first time, that cognitive quotients and pattern of functioning showed no change with age. These findings have subsequently been confirmed in other cohorts [2,3,4,5] and the assumption that cognitive function does not change over time has become commonly accepted. Hellebrekers et al. [6] reported a longitudinal follow-up study on verbal span and processing speed in Duchenne boys and found a developmental stagnation in verbal-span capacity, irrespective of normal processing speed, appearing more pronounced in patients missing Dp427 and Dp140. The concomitant involvement of Dp71 increases the risk of cognitive impairment and associated psychiatric comorbidities [3,4,5].

In the last decade there has also been an effort to better understand the mechanisms underlying the heterogeneity of the brain involvement in DMD and possible brain-related comorbidities [7,8,9]. Several studies have highlighted the association between cognitive function, site of DMD mutation, and involvement of different brain dystrophin isoforms [10,11,12]. While a few cross-sectional studies have reported comorbidities of cognitive and emotional aspects [13], the possible effect of behavioral patterns on changes in cognitive functioning has not been systematically explored.

The aim of our study was to evaluate the stability in intellectual functioning in DMD boys who had multiple assessments and the possible relationship to behavioral functioning and DMD genetic mutation.

## 2. Materials and Methods

All affected individuals were followed at the Child Neurology Unit and Nemo Center of the Fondazione Policlinico Universitario A. Gemelli IRCCS. Affected individuals were recruited and evaluated between 2010 and 2021. Boys were included if they had a genetically confirmed DMD diagnosis, and their age was between 3 and 17 years. As part of our clinical assessment, in agreement with care recommendations, we routinely perform a battery of cognitive and behavioral tests. Patients were included if they had at least two cognitive assessments at least 6 months apart. Boys with severe cognitive or behavioral problems who could not perform the Wechsler were analyzed separately.

Intellectual functioning was measured using the Wechsler scales: Wechsler Preschool and Primary Scale of Intelligence III edition (WPPSI-III) and Wechsler Intelligence Scale for Children III or IV edition (WISC-III and WISC-IV). Only total intelligence quotient (TIQ) scores were used for analysis purposes (mean = 100; SD = 15).

Behavioral issues were investigated through Child Behavior Checklist (CBCL: T scores: mean = 50; SD = 10), which is a 118-item questionnaire of internalizing and externalizing behaviors for the evaluation of children aged 6–18 years.

Attention deficits/hyperactivity symptoms were assessed by the psychologist with observation at the time of performing the other tests using the criteria from Diagnostic and Statistical Manual of Mental Disorders (DSM) IV or 5.

In order to assess possible differences related to the involvement of different brain dystrophin isoforms, the DMD cohort was subdivided into: patients with mutations expected to affect expression of Dp427 only (mutations upstream exon 44), patients with mutations expected to affect both Dp427 and Dp140 (mutations after exon 51), and patients in whom the DMD mutation was expected to affect all the isoforms, i.e., Dp427, Dp140, and Dp71 (mutations after exon 63). Mutations between 44 and 51 were reported separately, as the effect of these mutations on the involvement of Dp140 in these patients cannot be easily inferred from the mutation-site analysis. The study was approved by the Ethics Committee of our center.

Demographic and clinical characteristics were summarized using frequencies (percentage) for categorical variables and mean (SD) or median(first-third quartile) for continuous variables, unless otherwise stated. The patient population was subdivided into subgroups based on behavioral findings (normal results on both CBCL and attention tests; abnormal CBCL; attention deficits in patients with normal CBCL), IQ results (DSM 5 classification: normal IQ = 115–85, borderline intellectual functioning = 85–70, mild intellectual disability = 70–55, moderate intellectual disability < 55), and brain dystrophin involvement (Dp427 only, equivocal, Dp140 + Dp427, Dp71 + Dp140 + Dp427). For patients who had at least two assessments, we assessed whether the boys had stable TIQ results over time. This was measured using a cutoff of 11 points, as computed with the reliable-change method [14]. The non-parametric Kruskal–Wallis test was used in to compare differences in magnitude of changes overtime by behavioral findings, IQ results, and brain dystrophin isoform involvement. *p* value was set at <0.05.

## 3. Results

Of the 77 boys with DMD in our cohort, seven were unable to complete the tests and were classified as untestable. The remaining 70 patients had at least two assessments using the Wechsler scales and were included in the main analysis of the study: 29/70 had two assessments, 28 had three, 8 had four, and 5 more than four assessments for a total of 194 assessments in 70 patients. The time interval between assessments ranged from 6 months to 6 years (mean = 1.68, SD = 1.18).

Twenty-six of the 70 DMD boys had reduced expression of Dp427 only, 23 boys had Dp427 and Dp140 involvement (mutations after exon 51), and five patients had reduced expression of Dp427, Dp140, and Dp71 (mutations after exon 63) (Table 1). Sixteen had mutations between exons 44 and 51 and are reported separately. Of the seven untestable patients one had mutations before exon 44, five between 44 and 51, and one after exon 63.

### 3.1. Cognitive Tests

Forty-three patients had normal IQ, 18 had borderline intellectual functioning, 5 had mild intellectual disability, and 4 had moderate intellectual disability on their first assessment. The additional seven patients who were untestable at baseline continued to be untestable on several occasions. The TIQ for the whole cohort ranged between 40 and 120 (mean = 81.61; SD = 16.95).

There were 131 couples of consecutive assessments in the 70 patients with multiple assessments. Of the 131, 15 had two assessments at 6 months, 61 at 1 year, 32 at 2 years, 12 at 3 years, and 11 more than 4 years apart. There was no statistical difference in the magnitude or stability of changes between the subgroups (*p* > 0.05).

The mean TIQ changes in the cohort of 70 DMD boys ranged between −42 and 25 (mean = −2.99 points, SD = 12.29).

### 3.2. Behavioral Problems

Twenty-seven of the 70 boys with DMD had no behavioral disorders, 17 had abnormal CBCL total scores (two internalizing, two externalizing, 13 total score). Another 26 patients had clear signs of attention deficits despite this not being reported by the family in the CBCL.

Table 1 shows details of the brain dystrophin involvement in the different subgroups.

#### 3.2.1. Cognitive Changes and Behavioral Findings

**Figure 1:**
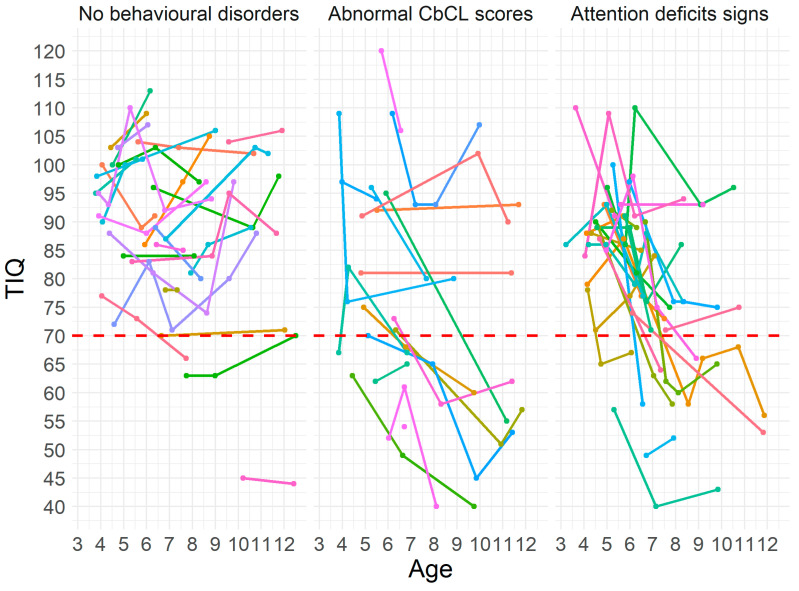
Individual IQ changes in patients with no behavioral disorders, with abnormal CBCL, and in those with attention deficits. Key to figure: dashed red line TIQ = 70, each colored line represent an individual patient.

There was a difference in the magnitude of changes among subgroups (*H*(2) = 10.32, *vp* = 0.006) with boys with no behavioral/attention disorder having smaller changes compared to those with attention (*p* = 0.004) and behavioral disorders (*p* = 0.012) (Figure 1).

**Figure 2:**
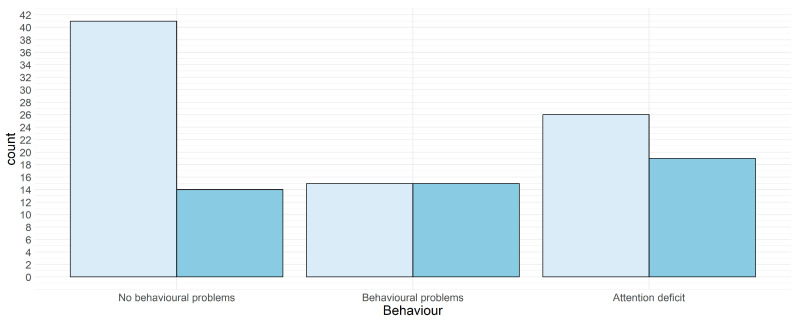
IQ changes (more or less 11 points) in patients subdivided according to behavioral findings. Key to figures: light blue < 11 points, blue ≥ 11 points.

A difference in quotients of more than 10 points was found in 47 of the 131 consecutive assessments (35.87%), this was statistically significant *X*2 (2, *N* = 131) = 10.964, *p* = 0.004 with patients without behavioral problems having fewer changes above the RC level (11 points) (Figure 2).

#### 3.2.2. Cognitive Changes and Cognitive Profile

**Figure 3:**
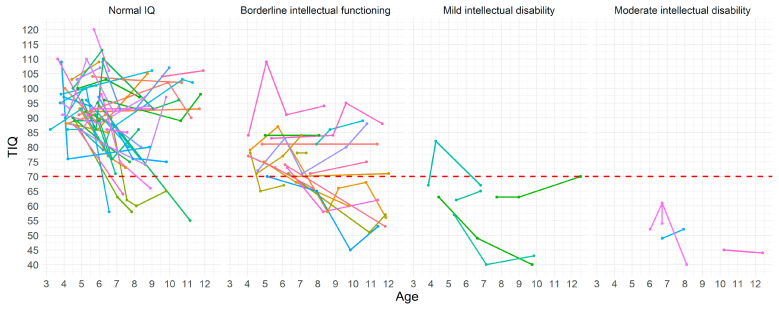
Individual IQ changes in patients with normal IQ, borderline intellectual functioning, mild intellectual disability, and moderate intellectual disability. Key to figure: dashed red line TIQ = 70, each colored line represent an individual patient.

The IQ changes in the group with normal IQ ranged between +24 and −42 (mean = −4.95; SD = 12.29) in the group with borderline intellectual functioning between +25 and −21 (mean = −0.11, SD = 12.5) in the group with mild intellectual disability between +15 and −21 (mean = −3.11, SD = 12.72), and in the group with moderate intellectual disability between +8 and −9 (mean = 2.37, SD = 13.23). There was no significant difference in the magnitude of changes among subgroups (*H(3*) = 4.98, *p* > 0.05) (Figure 3).

**Figure 4:**
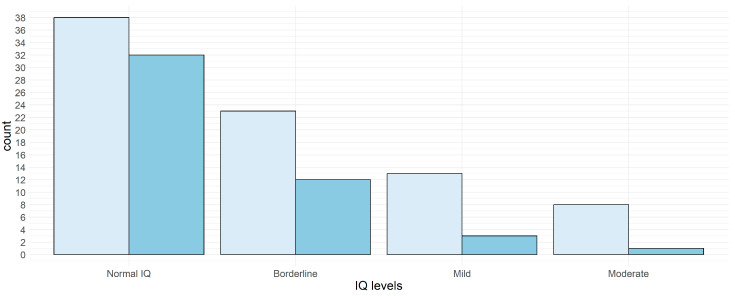
IQ changes (more or less 11 points) in patients subdivided according to cognitive profile. Key to figures: light blue < 11 points, bue ≥ 11 points.

A difference in quotients of more than 10 points was found in 47 of the 131 consecutive assessments (35.87%) (Figure 4), the difference among cognitive levels was not significant (*p* = 0.05).

#### 3.2.3. Cognitive Changes and Brain Dystrophins Involvement

**Figure 5:**
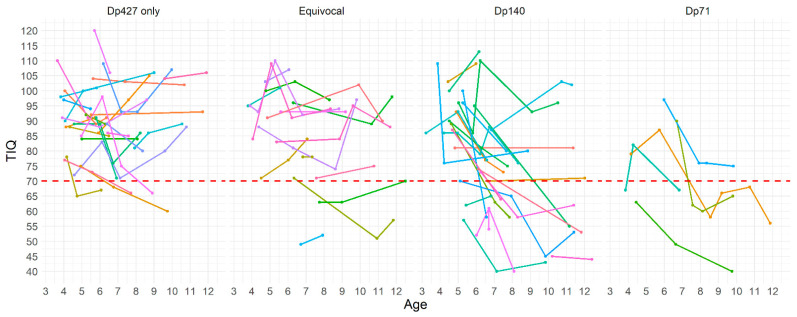
Individual IQ changes in patients subdivided according to brain dystrophin isoform involvement: Dp427 only, equivocal, Dp140, and Dp71. Key to figure: dashed red line TIQ = 70, each colored line represent an individual patient.

The IQ changes in the group with involvement of Dp427 only ranged between +14 and −23 (mean = −1.75, SD = 12.34); in the ‘equivocal’ group (mutations between exon 44 and 51), between +25 and −40 (mean = −2.51, SD = 12.45); in the group with definitive involvement of Dp 140, between +24 and −42 (mean = −5.09, SD = 12.85) and in the group with additional involvement of Dp71 between +15 and −29 (mean = −5.91, SD = 14.15). There was no difference in the magnitude of changes among subgroups (*H(3*) = 5.50, *p* > 0.05) (Figure 5).

**Figure 6:**
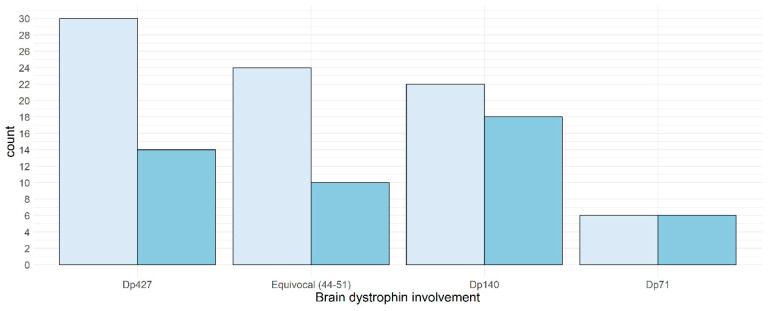
IQ changes (more or less 11 points) in patients subdivided according brain dystrophin involvement. Key to figures: light blue < 11 points, blue ≥ 11 points.

A difference in quotients of more than 10 points was found in 47 of the 131 consecutive assessments (35.87%): the difference between brain dystrophin subgroups was not significant (*p* > 0.05) (Figure 6).

## 4. Discussion

Several cross-sectional studies have previously reported that cognitive function does not significantly change over time [2,3,4,5]. Longitudinal assessments in our cohort confirm that the mean changes in a large cohort of 70 DMD boys was relatively small (−2.99 points). The standard deviations were however much larger (12.29); these reflecting the wide variability of the changes. Approximately a third of the 131 consecutive cognitive assessments showed a difference of more than 11 points with changes in IQ up to 42 points. When we analyzed the magnitude of changes in relation to different variables, such as presence of behavioral/attention disorder, site of mutation, or cognitive status at baseline, we found that the presence of behavioral or attention disorder was more related to larger TIQ changes. Affected individuals with normal behavior had higher and overall more stable TIQ over time and, with few exceptions, they were all within the normal/borderline range while patients with attention deficits or behavioral disorders more often had more variable results. Changes above 11 points were observed in 33% of the consecutive assessments performed in boys with abnormal CBCL and in 45% of those with attention deficits.

In contrast, the magnitude of IQ changes over time was not significantly related to the level of cognitive function or to the involvement of brain dystrophin isoforms. The risk of showing larger changes was higher in the patients with involvement of Dp140 or Dp71 compared to those with involvement of Dp427 only (40% and 17% vs. 24%), but this was not statistically significant.

One of the advantages of our study is that the assessments were performed as part of our clinical routine and by the same examiner and generally performed before the motor function assessments. The study however had several limitations as, because of its retrospective nature, the assessments were not always performed systematically at regular intervals. This partly reflects the fact that in the last few years, with increasing evidence of global or selective cognitive difficulties, we intensified the frequency of routine cognitive assessments compared to previous years [7]. In addition, different versions of the Wechsler Scales (WPPSI-III, WISC-III, and WISC-IV) were compared mutually.

Because of these factors, we selectively chose to focus on changes in the total IQ in order to detect possible major variations over time that should have not been grossly affected by the version of the scale used.

Our results succeeded in highlighting that despite mean changes being small, large changes in individual patients were not infrequent. The analysis of the results raised a number of questions. The increased rate of changes in patients with behavioral/attention issues makes one wonder whether, in these boys, the TIQ changes reflect true changes in cognitive function rather than a different level of attention and collaboration at different testing times. Our observation highlights the need to have strict standard operation procedures on the modalities of testing in order to reduce the risk of reduced attention leading to a poor performance. Our findings also highlighted a discrepancy between the CBCL and the assessment of attention performed by the psychologists. In 27 boys (37%), the psychologists reported obvious signs of attention deficits during the assessments that did not match with the normal attention reported by the parents on the CBCL items. The psychologist observations, based on the DSM-IV/V criteria, were always in line with reports of attention deficits from the school. An ongoing study using the Conners scales for both parents and teachers confirms the higher detection by teachers and the lower rate of abnormal responses by the caregivers. These findings support previous evidence that the parent-reported CBCL may be of limited value in DMD, highlighting the need to perform additional structured assessments to identify possible attention deficits that may not be acknowledged by the parents [15].

In conclusion, our results suggest that reliable changes in TIQ may occur in DMD boys and these are more likely to be associated with behavioral attention changes than with the involvement of Dp140 and 71 or level of intellectual disability. These findings may be explained by the fact that behavior and attention problems are not always associated with low IQ or with involvement of Dp140 and 71 [11,16] and suggest that particular attention should be paid to these aspects that are increasingly recognized and treated in DMD boys [17,18,19]. Another limitation of the study is that it was performed over a relatively long period of time during which the version of the Wechsler scales changed (e.g., WISC III to WISC IV). Consequently, we could not perform a comparison of subgroup analysis or use some of the tools included in the more recent versions.

This is the first study to systematically report data on follow-up of intellectual functioning over time. Further prospective studies systematically assessing both cognitive and behavioral aspects using structured assessments including school reports on academics are needed to better understand the relationship between these aspects and their stability and impact on intellectual function over time.

## Figures and Tables

**Table 1:** Details of the brain dystrophin involvement in the different subgroups of intelligent quotients (IQ) levels, behavioral findings, and brain dystrophin involvement.

		Normal IQ	BIF	MID	MoID
No behavioral disorders (N)	Dp427 only	9	4	0	0
	Equivocal	6	2	1	0
	Dp140	3	1	0	1
	Dp71	0	0	0	0
Behavioral disorders (N)	Dp427 only	4	1	0	0
	Equivocal	1	1	0	0
	Dp140	3	3	1	1
	Dp71	0	0	2	0
Attention deficits (N)	Dp427 only	6	1	0	1
	Equivocal	1	3	0	1
	Dp140	8	1	2	0
	Dp71	2	1	0	0

BIF, borderline intellectual functioning; MID, mild intellectual disability; MoID, moderate intellectual disability.

## Data Availability

Data are available on request.
